# The tymbal muscle of cicada has flight muscle-type sarcomeric architecture and protein expression

**DOI:** 10.1186/s40851-017-0077-4

**Published:** 2017-09-01

**Authors:** Hiroyuki Iwamoto

**Affiliations:** Japan Synchrotron Radiation Research Institute, SPring-8, 1-1-1 Kouto, Sayo-cho, Sayo-gun, Hyogo 679-5198 Japan

**Keywords:** Tymbal muscle, Cicada, Insect flight muscle, Troponin, Synchrotron radiation, X-ray diffraction

## Abstract

**Background:**

The structural and biochemical features of the tymbal (sound-producing) muscle of cicadas were studied by X-ray diffraction and immunochemistry, and compared with those of flight muscles from the same species.

**Results:**

The X-ray diffraction pattern of the tymbal muscle was very similar to that of the dorsal longitudinal flight muscle: In both muscles, the 2,0 equatorial reflection is much more intense than the 1,1, indicating that both muscles have a flight muscle-type myofilament lattice. In rigor, the first myosin/actin layer line reflection was finely lattice-sampled, indicating that the contractile proteins are arranged with a crystalline regularity as in asynchronous flight muscles. In contrast, the diffraction pattern from the tensor muscle, which modulates the sound by stressing the tymbal, did not show signs of such high regularity or flight muscle-type filament lattice. Electrophoretic patterns of myofibrillar proteins were also very similar in the tymbal muscle and flight muscles, but distinct from those from the tensor or leg muscles. The antibody raised against the flight muscle-specific troponin-I isoform reacted with an 80-kDa band from both tymbal and flight muscles, but with none of the bands from the tensor or leg muscles.

**Conclusion:**

The close similarities of the structural and biochemical profiles between the tymbal and the flight muscles suggest the possibility that a set of flight muscle-specific proteins is diverted to the tymbal muscle to meet its demand for fast, repetitive contractions.

**Electronic supplementary material:**

The online version of this article (10.1186/s40851-017-0077-4) contains supplementary material, which is available to authorized users.

## Background

Both body and visceral muscles of insects are cross-striated, having sarcomeric structures. Flight muscles, especially those of bees and flies have highly specialized functions; i.e., they are asynchronous (there is no one-to-one correspondence between motor nerve impulses and wing beats) and are characterized by the function of stretch activation [1, 2]. The asynchronous flight muscle has a highly specialized regular structure ([3] and references therein). More primitive winged insects have synchronous flight muscles (one nerve impulse elicits a single wing-beat), but their structures are more specialized than those of body muscles.

Cicadas are among the insects with synchronous flight muscles, and are exhibit the capacity of producing loud songs. Such sounds are generated by a structure called the tymbal, a chitinous plate located on both sides of the first abdominal segment. A tymbal usually has a number of ribs, which produce a series of clicking sounds when it is deformed by a single twitch of the tymbal muscle. Most of the abdominal volume of a male cicada is filled with air, and the entire abdomen acts as a resonator [4–6].

**Fig. 1:**
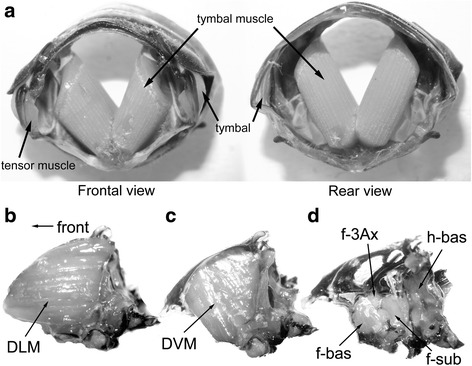
Photographs tymbal muscles and flight muscles. **a** tymbal muscles and related structures, housed in the first abdominal segment of *Graptopsaltria nigrofuscata*. **b**-**d** Flight musculature of *Meimuna oparifera*. The entire thorax is split along the midline and viewed from inside. **b** When the thorax is split along the midline, the massive dorsal longitudinal muscle (DLM) is observed. **c** The DLM has been removed to reveal the massive dorso-ventral muscle (DVM). **d** The DVM has also been removed to reveal the direct flight muscles (only the flight muscles relevant to this paper are annotated). f-bas, forewing basalar; f-3Ax, forewing 3Ax muscle (steering muscle), f-sub, forewing subalar; h-bas, hindwing basalar. The hindwing subalar lies behind the hindwing basalar

**Fig. 2:**
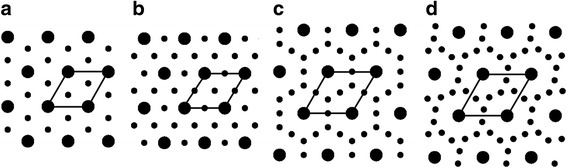
Types of myofilament lattice of muscle. **a** 1:2 lattice (the myosin-to-actin filament number ratio is 1:2), seen in vertebrate skeletal and cardiac muscles. **b** 1:3 lattice, seen in insect flight muscles. **c** 1:5 lattice; **d** 1:6 lattice. These are seen in non-flight muscles of insects and in muscles of other arthropods. See also [19, 37]. The larger dots represent the myosin filaments, and the smaller dots, the actin filaments. The area marked by the lines represents the unit cell, which contains one myosin filament

The tymbal muscle is a V-shaped, conspicuous presence in the first abdominal segment (Fig. 1), and its developmental/evolutional origin is believed to be the dorsoventral muscle associated with each segment [5]. Its visual appearance (color, fiber diameter, etc.) is similar to that of major flight muscles. Its ultrastructure has been investigated using electron microscopy [7–9] in the species *Platypleura capitata*, *Cyclochila australasiae*, and *Magicicada cassini*. Micrographs have shown that the myofilament lattice structure of the tymbal muscle is identical to that of major flight muscles, i.e., the myosin: actin filament number ratio is 1:3 and an actin filament is located midway between two neighboring myosin filaments (Fig. 2). Functionally, tymbal muscles are generally synchronous; nine genera of cicadas are proven to have synchronous tymbal muscles [8]. However, the tymbal muscle of an oriental species, *Platypleura capitata* is inferred (although not directly demonstrated) to be asynchronous [8], as are the flight muscles of higher-order insects. It would be very peculiar for an asynchronous muscle to occur elsewhere, while the flight muscles remain synchronous. Although the wing-beat frequencies of cicadas are in the range manageable by synchronous flight muscles (30–50 Hz [8]), having asynchronous tymbal muscles may be beneficial for supporting much higher sound vibration frequencies (234–389 Hz [8]). Nonetheless, the contraction frequency of the tymbal muscle is still expected to be below 100 Hz due to the multiple sound-producing mechanism described above.

Here we used X-ray diffraction and immunochemistry to study the flight and tymbal muscles of a number of cicada species, to obtain further insights into the structure and function of these muscles. The species used include *Platypleura kaempferi*, closely related to *P. capitata*. In addition to the filament number ratio, X-ray diffraction can provide many other pieces of structural information about the sarcomeric structure, such as the helical symmetry of myofilaments and the regularity of protein arrangement, and their changes under different physiological conditions (for review see [10]). Notably, asynchronous flight muscles of the giant waterbug and other species are known to exhibit a crystalline order of contractile proteins, a feature that manifests as discrete reflection spots in the diffraction pattern ([11] and references therein). This point is important, as X-ray diffraction may discriminate between the asynchronous tymbal muscle (if it is truly so) and the flight muscle of cicadas (however note that no report to date has demonstrated that the latter is asynchronous). The contractile proteins were characterized by gel electrophoresis and immunoblotting, using an antibody (MAC143) raised against the flight muscle-specific isoform of troponin-I (also called troponin-H because of its unusually large molecular mass [12]). Its large molecular mass is due to its long Pro-Ala-rich C-terminal extension, to which the antibody is expected to react. In Diptera, the sequence is known to have translocated to the C-terminus of tropomyosin [13–16]. The antibody is known to react with the flight muscle proteins of all insect species examined to date, irrespective of whether they are asynchronous [17].

In addition, some mechanical measurements were made on the skinned muscle fibers of the flight and tymbal muscles, to test whether they exhibit stretch-activation, a characteristic mechanical feature of an asynchronous flight muscle.

## Results

In the present study, muscle preparations were obtained from several cicada species, and this is mainly due to the limited availability of each species. Generally the basic molecular architecture, such as the sarcomeric structure, does not vary among species in a single family, or even within a single order of animals. Thus, the diffraction patterns and immunochemical results from cicada muscle fibers are all expected to be similar for the three species used. Because of this, only the results from *Meimuna oparifera* are described here unless otherwise stated (for the results of other species see Additional files 1, 2, 3 and 4).

### X-ray diffraction patterns

**Fig. 3:**
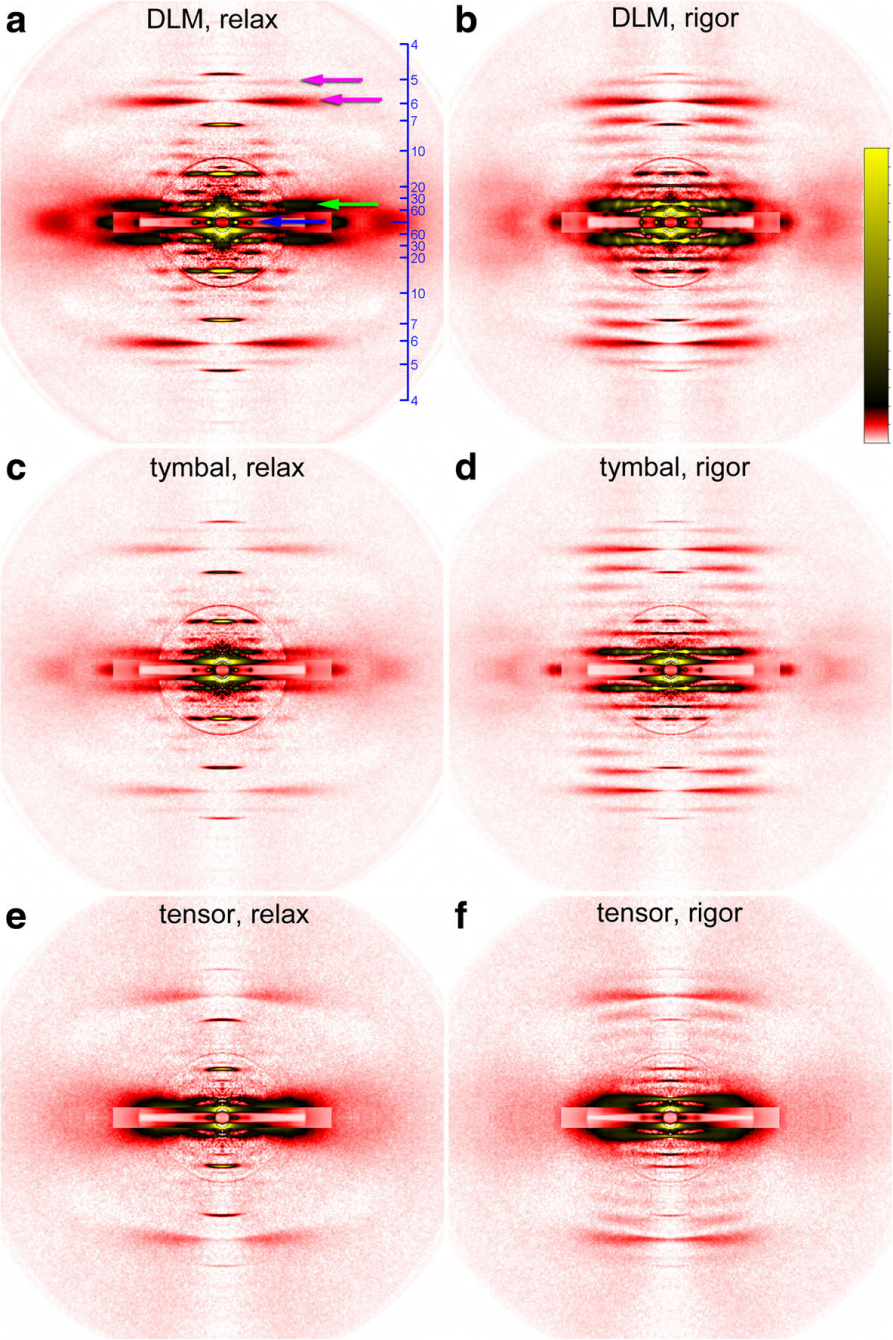
X-ray diffraction patterns of the flight and tymbal muscles of *Meimuna oparifera.* (**a**) and (**b**), DLM; (**c**) and (**d**), tymbal muscle; (**e**) and (**f**), tensor muscle. **a, c** and **e** were recorded in the relaxed state, and **b**, **d** and **f**, rigor state. Blue arrow, equatorial reflections; green arrow, first myosin/actin layer line reflection, magenta arrows, actin layer line reflections. The scale on the right of A represents the *d*-spacing in nm. Note that in rigor, the actin layer lines are enhanced, and the first myosin/actin layer line is finely lattice-sampled in flight and tymbal muscles but not in tensor

**Fig. 4:**
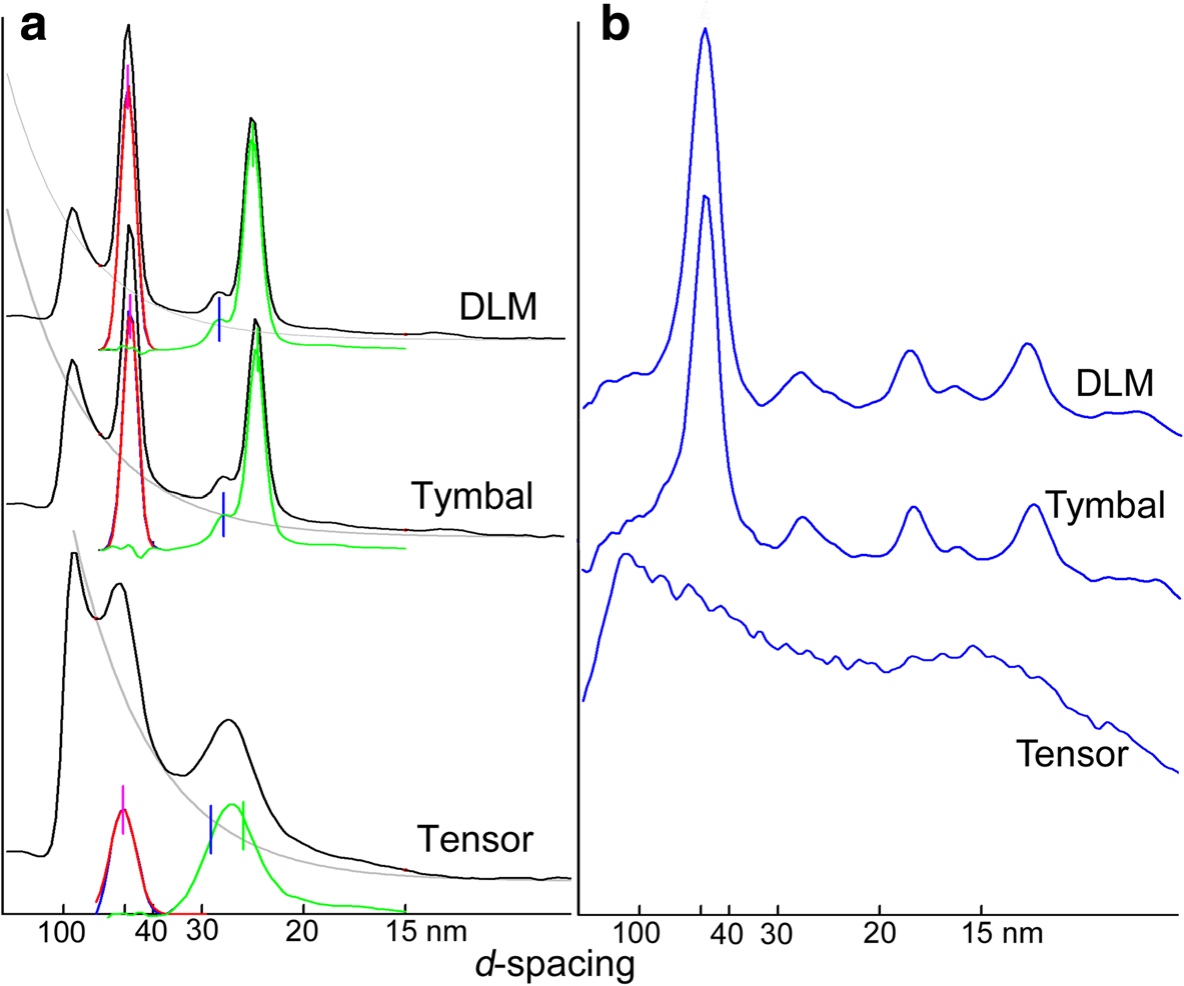
Intensity profiles of the equatorial (**a**) and the first myosin/actin layer line (**b**) reflections, taken from the diffraction patterns from *Meimuna oparifera* in Fig. 3. In **a**, the red curve is the Gaussian fit of the 1,0 reflection after subtraction of the background, approximated as a single exponential decay function (gray). The blue curves (almost hidden underneath the red curves) are the observed intensity profile after subtraction of the background. The green curve is the residual remaining after the Gaussian fit of the 1,0 is further subtracted. The red vertical bar is the center of the Gaussian fit of the 1,0, and the blue and green vertical bars are the positions of the 1,1 and 2,0 reflections, respectively, calculated from the position of the 1,0 reflection. The leftmost peak is the edge of the beamstop. Note that the 2,0 reflection is much more intense than the 1,1 in flight and tymbal muscles except for the tensor

The X-ray pattern recorded from the muscles of *Meimuna opariferea* are shown in Fig. 3. The patterns in Fig. 3a and b were taken from the indirect flight muscle (dorsal longitudinal muscle, DLM). Although the cicada flight muscles are synchronous, the arrangement of the contractile proteins of the DLM is fairly regular, as is evident from the spot-like appearance of the 1st actin/myosin layer line reflection (layer-line reflections arise from the helical arrangement of actin or myosin molecules on the myofilaments) (see also Fig. 4b). This spot-like appearance is called lattice sampling, and the intensity is observed only in the position indexable to the planes of the myofilament hexagonal lattice. Lattice sampling is conspicuous in asynchronous flight muscles, and also often occurs in higher-order layer-line reflections.

In the DLM, the lattice sampling is already present in the relaxed state (Fig. 3a), but is more pronounced in rigor (Fig. 3b), indicating that the rigor linkage between the actin and myosin filaments increases the regularity of protein arrangement in the sarcomere. The lattice sampling is much weaker in other insects with synchronous flight muscles, such as lepidopterans (unpublished).

In the equatorial reflections of the DLM, the 2,0 reflection is more intense than the 1,1 reflection (Fig. 4a), indicating that this muscle has a flight-muscle type lattice structure (myosin: actin filament number ratio = 1:3; see Fig. 2 and [18]) as in asynchronous flight muscles.

Although not recorded from *M. oparifera*, the diffraction patterns from the basalar muscle from other species (Additional file 1: Figure S1 & Additional file 3: Figure S3) are very similar to those from the DLM; the 1st layer line reflection is sampled in the same way, and in the equator, the 2,0 reflection is stronger than the 1,1. Therefore it is considered to have the same sarcomeric architecture as the DLM.

The diffraction patterns from the tymbal muscle are also very similar to those from the DLM (Fig. 3c, d). The 1st layer line is clearly lattice-sampled (Fig. 4b), and the 2,0 is stronger than the 1,1, indicating that it also has a flight muscle-type filament lattice (Fig. 4a). This is also true for other cicada species, including *P. kaempferi* (see Additional files 1, 2, 3 and 4). This confirms observations from electron microscopy (for references see Introduction). Although the tymbal muscle of *P. capitata* (a species closely related to *P. kaempferi*) is inferred to be asynchronous [8], the lattice sampling in the layer line from *P. kaempferi* is limited to the first layer line, and there is no evidence that the structural regularity of the tymbal muscle is higher than that of the synchronous DLM (Additional files 1: Figure S1). The values of *d*-spacing for the 1,0 lattice plane in rigor were very similar for the three muscles. They were 47.9, 47.2, and 47.8 nm for the DLM, basalar (*P. kaempferi*) and tymbal, respectively.

Diffraction patterns were also taken from the tensor muscle (Fig. 3e, f). The tensor muscle is to increase the curvature of the tymbal, thus modulating the tone of the cicada songs [4]. Unlike in the flight muscles and the tymbal muscle, the lattice sampling on the first layer line is not evident (Fig. 4b), indicating a low structural regularity. In the equator, the 1,1 and 2,0 reflections are not separated, but it is evident that the intensity of the 2,0 is comparable to that of the 1,1 (Fig. 4a), suggesting that its lattice structure is of body-muscle type (1: 5 or 1: 6), not the flight muscle type (1: 3) (Fig. 2). The *d*-spacing for the 1,0 plane was 49.5 nm and was apparently greater than those for flight and tymbal muscles, in agreement with the 1:5 or 1:6 lattice that has a greater *d*-spacing than the 1:3 lattice [19].

### Immunochemistry

**Fig. 5:**
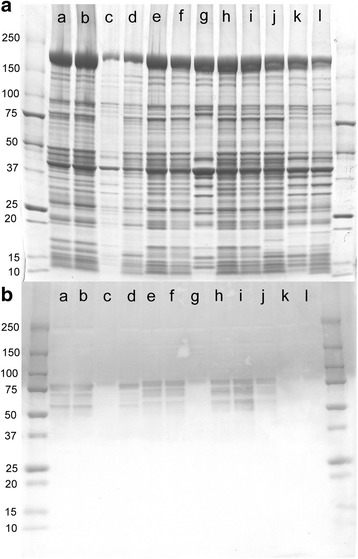
SDS gel electrophoretic and immunoblot patterns of the muscle fibers from *Terpnosia vacua* and *Meimuna oparifera.* **A** Coomassie brilliant blue-stained SDS gel electrophoretic pattern; **B** Western blot pattern obtained by using an antibody against flight muscle-specific troponin-I (troponin-H). Lanes a-d, *Terpnosia vacua*; lanes e–l, *Meimuna oparifera*. a and e, DLM; b and f, DVM; c and g, 3Ax; d and j, tymbal; h, basalar; i, subalar; k, tensor; l, leg (foreleg femur). The patterns were corrected for the deformation of the gels (expansion and fanning out) (applies also to Additional file 3: Figure S3)

Samples were taken from flight, tymbal and other non-flight muscles, and were subjected to SDS-PAGE and immunoblotting. The CBB-stained SDS-PAGE pattern and the result of immunoblotting are shown in Fig. 5a and b, respectively (samples of *Terpnosia vacua* were loaded on the same gel). The patterns for the flight muscles, including indirect DLM and DVM, and direct basalar and subalar muscles, are identical (except for the 3Ax muscle, also known as the wing-folding muscle), indicating that an identical set of protein isoforms are expressed in these muscles. They all have a ~ 80 kDa band that cross-reacts with MAC143, except for the 3Ax, and this pattern of reaction is identical to that for a beetle [19]. MAC143 is an antibody raised against the flight muscle specific troponin-I (troponin-H) isoform (Fig. 5b). Some lower molecular weight bands (fainter in the CBB-stained pattern) also cross-reacted with this antibody, and they may be splicing variants or degraded products. The SDS-PAGE pattern for the tymbal muscle is very similar to those of flight muscles, except for some low-molecular weight bands between 15 and 20 kDa. Its 80-kDa band also cross-reacts with MAC143. The patterns for the 3Ax muscle, tensor and leg muscles are similar to each other, and they lack the 80-kDa band that cross-reacts with MAC143. The tymbal muscle specimens from other cicada species also had a ~ 80 kDa band that reacted with MAC143 (Additional file 4: Figure S4).

The muscle types of as determined by X-ray diffraction and immunochemistry are summarized in Table 1.

**Table 1:** Summary of muscle types in four cicada species examined in this study

	Indirect flight muscles		Direct flight muscles			Body muscles		
Species	DLM	DVM	Bas	Sub	3Ax	Tym	Ten	Leg
*Terpnosia vacua*	F/f	f			b	F/f		
*Meimuna oparifera*	F/f	f	f	f	b	F/f	B/b	b
*Platypleura kaempferi*	F		F			F		
*Graptopsaltria nigrofuscata*	F/f	F/f	F/f	F/f	b	F/f	b	b

F or f, flight muscle type (1:3 myofilament lattice and sampled 1st layer line, expression of flight muscle-specific troponin I isoform); B or b, body muscle type (1:5 or 1: 6 lattice and non-sampled 1st layer line, lack of flight muscle-specific troponin I isoform). Capital letters, evidence from X-ray diffraction; small letters, evidence from immunoblotting. Bas, basalar; Sub, subalar, Tym, tymbal, Ten, tensor

### Mechanical measurements

**Fig. 6:**
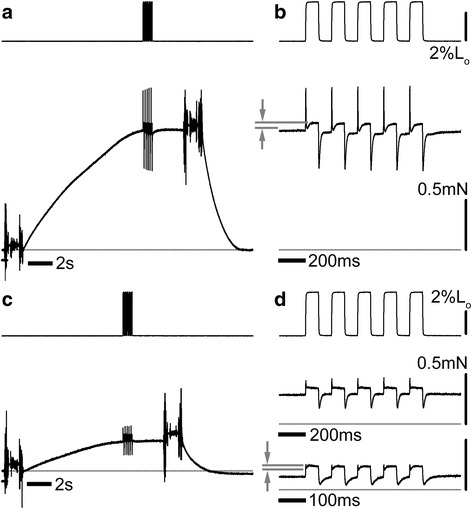
Mechanical response to the repeated stretches/releases of the calcium-activated DLM (**a, b**) and the tymbal (**c, d**) muscle fibers from *Platypleura kaempferi.* (**a**) and (**c**), slower time base; (**b**) and (**d**), faster time base. Upper trace, length (stretch upward); lower trace, force. In **d**, the responses to both 5-Hz and 10-Hz repetitions are indicated. The gap between the two horizontal gray lines (indicated by arrows) is the amplitude of the stretch-activated force

Mechanical measurements were performed only for *P. kaempferi*, to test if its tymbal muscle fibers exhibit any characteristics of asynchronous muscle. Isolated glycerinated muscle fibers from the DLM or the tymbal muscle were activated at pCa = 4.0 and step stretches (amplitude, 2–3% of the just-taut length, L_o_) were applied (Fig. 6). Although this amplitude of stretch was greater than usually needed to elicit fully stretch-activated forces in asynchronous flight muscle fibers (e.g., [20, 21]), it elicited only a small stretch-activated force in cicada DLM fibers (Fig. 6b, gray arrows). This is very similar to the responses to stretch of other synchronous flight muscles [17]. The mechanical response of the tymbal muscle fibers was not very different from that of the DLM (Fig. 6d). At the repetition rate of 5 Hz (Fig. 6d, middle trace), there was no obvious stretch-activated force, but at 10 Hz, a small stretch-activated force was recognized (Fig. 6d, bottom trace), and its rate of rise was faster than that for the DLM (half rise time = 7.4 ms vs. 20.2 ms for DLM). This may indicate that the contractile proteins of the tymbal muscle exhibit faster kinetics than those of the DLM.

## Discussion

In this study, the structural, biochemical and mechanical features of the tymbal muscle of cicada were compared with those of the major flight muscle. The results show that, with regard to all three aspects, the tymbal muscle is very similar to (but not identical with) the major flight muscle.

### Fine structure

Electron microscopy studies have shown that the tymbal muscle has a flight muscle-type myofilament lattice arrangement [7–9]. The most important finding is that the arrangement of the contractile proteins in the sarcomere of both flight and tymbal muscles is considerably regular, as evidenced by the crystalline sampling of the first myosin/actin layer line reflection, especially in rigor. This observation agrees with the previous end-on diffraction studies of myofibrils, showing that in both flight and tymbal muscles of cicadas, long-range crystallinity (i.e., the lattice plane orientation is preserved along the entire length of the myofibril) was observed [22]. This is unusual for insect groups with synchronous flight muscle. Also, the tymbal muscle is the first example of non-flight insect muscle that is demonstrated to have such high structural regularity.

In the present study the diffraction patterns were also recorded from the basalar muscle, one of the direct flight muscles. The basalar and subalar muscles of the cicadas are not well-developed, and it is unlikely that they provide actual driving force of the wings. Nevertheless, the basalar of the cicada is shown here to have the same structure as the DLM. In beetles, in which the basalar is very well developed and is stretch activatable [2], both basalar and subalar muscles are shown to have structures identical to those of the DLM and DVM [19].

### Biochemical features

Troponin-I, one of the three troponin subunits, usually has a molecular mass of ~25 kDa. However, the flight muscle-specific troponin-I isoform (troponin-H) has a long Pro-Ala-rich C-terminal extension [12], and because of this, its apparent molecular mass on the gel is 75–80 kDa. The antibody against this extension is known to cross-react with the proteins from all winged insect groups examined, synchronous or asynchronous [17], and in Diptera, this extension is found in the C-terminus of tropomyosin, instead of troponin I [13–16]. The extension has repeating GESGAGKT motifs or the like, and it exists in the most primitive winged insects [17]. The fact that this extension exists in tropomyosin in all of Dipterans, a monophyletic group, suggests that the sequence has translocated from troponin-I from tropomyosin in the ancestral from of Diptera. The role of the extension is still to be investigated, but in fruit fly, it is known to bind γ-glutathione S-transferase (GST [15]), and in bumblebee, the enzymatic removal of the extension leads to a loss of flight muscle-type 1:3 lattice integrity [23]. The GST that binds to the extension is suggested to have a protective role against deleterious effects of oxidative stress due to the high mitochondrial activity of the flight muscle [24]. The extension is not essential for the mechanical function of flight muscle [18, 25].

Here we have shown that the cicada flight muscles also express the 80-kDa protein, except for the 3Ax muscle. An important finding here is that the 80 kDa protein is also expressed in the tymbal muscle. Initially, the tymbal muscle was thought to be of flight-muscle origin, but later it was rather regarded to be a specialized form of the dorso-ventral muscle that exists in every abdominal segment [5]. It is possible that fast vibrational contractions are required for both flight and sound production, and it is beneficial to express the flight muscle-specific proteins in the tymbal muscle as a result of evolutional adaptation. The homopteran insects are generally known to communicate with vibrations (for smaller species they are not airborne but are transmitted by substrates such as leaves; e.g., [26]), and in a planthopper *Nilaparvata lugens*, the dorso-ventral muscle of the first abdominal segment (responsible for vibration) is known to express another flight muscle-specific protein, flightin [27].

### Mechanical properties

Although the flight muscles of cicadas are synchronous, the possibility of asynchrony has been suggested for *Platypleura capitata* [8]. This would be an interesting first example of synchronous and asynchronous versions of an identical muscle occurring in a single family of insects, and if such is the case, a higher structural order and a greater capacity of stretch activation would be expected for the asynchronous version. Here we compared the structure and function of the flight and tymbal muscles of closely related *P. kaempferi*, but their fine structure are found to be very similar, and no conspicuous stretch activation was observed in the tymbal muscle fibers. Asynchronous flight muscle fibers usually show higher resting stiffness, because of the well-developed C-filament that connects between the thick filaments and the Z-line. However, the tymbal muscle fibers of *P. kaempferi* showed only low resting stiffness (data not shown). Therefore, there is no evidence that suggest the asynchrony of its tymbal muscle.

The report that the tymbal muscle of a Brazilian cicada, *Fidicina*, is stretch-activatable presents a puzzling case [7]. The experiments were performed in a phosphate-buffered solution that is known to increase the stretch-activated force in vertebrate skeletal muscle fibers [28]. Therefore it is unclear whether there is a real discrepancy between their and our results.

### Evolution of muscle structure and function in cicada and related insects

The order Hemiptera is unique in that it contains both species with asynchronous and synchronous flight muscles. Synchrony/asynchrony of the flight muscles within Hemiptera has been extensively studied by Cullen [29], purely on the structural basis.

According to this study, the species belonging to suborder Heteroptera (true bugs) have exclusively asynchronous flight muscles, while suborder Auchenorrhyncha (containing cicada-like insects with highly variable body sizes) is a mixture of families with synchronous and asynchronous flight muscles. The flight muscles of cicadas (Cicadidae) are classified as synchronous, while the flight muscles of leafhoppers (Jassidae or Cicadellidae) are classified as asynchronous. This is supported by our observation that the flight muscle of a Cicadellid, *Bothrogonia*, is highly crystalline (Iwamoto et al., 2006 [22]), and is unmistakably stretch-activatable (unpublished).

The question is, synchronous cicadas or asynchronous Cicadellids, which come first in the course of evolution. Insects of suborder Auchenorrhyncha are generally known to communicate by vibration [26], and for this Cicadellids have a “striated tymbal homologous to that of cicadas”, and a set of less-specialized (as compared with cicadas) dorsoventral muscles are the main sound-producing muscles [5]. As discussed by Pringle [5], the missing link that connects Cicadellid insects and cicadas may be the primitive Australian cicadas of the genus *Tettigarcta*, which lacks the resonant air sac [5] but communicates via substrate-borne vibrations [30]. If modern cicadas occurred from small, asynchronous Cicadellid-like insects through the stage of *Tettigarcta*, this means that the asynchrony was lost at some point of body size enlargement, as the reduced wing-beat frequency does not require asynchrony. At the same time, they may have developed a resonant air sac for air-borne communication, for which the tymbal muscle accordingly became specialized. This evolutionary process would explain why the structure of the cicada flight muscle is more regular than that of the synchronous flight muscles in other insect orders. Because the set of genes for ordered sarcomeric structure is already present, it may be readily diverted to the tymbal muscle.

According to Curren [29], insects belonging to some other families of Auchenorrhyncha smaller than cicadas, such as Cercopoidea and Membracidae, also have synchronous flight muscles. Although this old study, based solely on structural characteristics, should be re-evaluated, it would not be surprising that they are synchronous, as they are generally larger than the average Cicadellids and their wing-beat frequencies are probably below 100 Hz. It would be interesting to know if they also have evolved from asynchronous ancestors.

In his lecture in 1980, Pringle [31] stated “One thing which does seem to be clear is that once the asynchronous mechanism had developed in a group of insects, the reverse evolution never occurs”. If the evolutionary scenario described above is correct, however, it would represent the first known example of such reverse evolution.

## Conclusion

The present study reveals that the sarcomeric structure of the cicada tymbal muscle is as regular as that of the flight muscle, and the tymbal muscle expresses the flight muscle-specific troponin-I isoform. The close similarities of the structural and biochemical profiles between the tymbal and the flight muscles suggest a possibility that a set of flight muscle-specific proteins are diverted to the tymbal muscle to meet the demand for its fast, repetitive contractions.

## Methods

### Materials

Four species of cicadas (*Terpnosia vacua*, *Meimuna oparifera*, *Platypleura kaempferi* and *Graptopsaltria nigrofuscata*) were collected in or near the campus of SPring-8. Flight muscle and other muscles were isolated from these insects, and were stored in a 50% mixture of glycerol and a relaxing solution at −20 °C until use. A few days before X-ray recordings, bundles of 3–4 muscle fibers were isolated from each muscle, and seven bundles were mounted on a pair of ceramic chips, as described previously [32].

### Solutions

For X-ray measurements, the muscle fibers were placed either in a relaxing solution or a rigor solution, and their compositions are basically the same as in previous studies [33, 34]. The relaxing solution contained 80 mM K-propionate, 20 mM imidazole, 10 mM EGTA, 4 mM ATP, 5 mM MgCl_2_, 20 mM phosphocreatine, and 400 U/ml creatine phosphokinase (C3755, Sigma-Aldrich) (pH = 7.2). The rigor solution contained 120 mM K-propionate, 20 mM imidazole, and 5 mM each of EDTA and EGTA (pH = 7.2). For mechanical measurements, the fibers were activated in the presence of calcium (10.1 mM total, pCa = 4.0) in addition to the components of the relaxing solution. Prior to activation, the fibers were immersed in a pre-activating solution with a reduced concentration of EGTA (0.5 mM).

### X-ray diffraction recordings

Static X-ray diffraction patterns were recorded at the BL45XU beamline of SPring-8 [35]. The detector was a cooled CCD (charge-coupled device) camera (C4792–98, Hamamatsu Photonics) in combination with a 6-in. image intensifier (VP5445-MOD, Hamamatsu Photonics). The exposure time was 2 s, and up to 40 diffraction patterns were recorded from a single set of muscle fiber bundles. The fibers were moved along their fiber axis by 100 μm after each exposure to reduce radiation damage. Diffraction patterns were first taken in the relaxing solution, and then in the rigor solution at 5 °C). The diffraction data were acquired by using the program HiPic (Hamamatsu Photonics). The diffraction patterns taken from the same set of fiber bundles were summed, and the four quadrants were averaged, and the background scattering was subtracted as described previously [20, 36].

### Gel electrophoresis and immunochemistry

The muscle samples were subjected to sodium dodecyl sulfate-polyacrylamide gel electrophoresis (SDS-PAGE) by using 5–20% gradient gels (Atto Corporation) and the bands were stained with Coomassie brilliant blue (CBB). Very approximately 0.1 mg of tissue was loaded to each lane for CBB staining, after the samples were heated to 95 °C in a sample buffer containing SDS. For immunoblotting, the amount of sample in each lane was 1/10 of that for CBB staining, and an antibody raised against flight muscle-specific troponin I (troponin H) (MAC143, Abcam, 1:5000 dilution), and the reaction was detected by using an anti-rat secondary antibody conjugated with alkaline phosphatase (A6066, Sigma-Aldrich, 1:5000 dilution) and a BCIP/NBT solution (B6404, Sigma-Aldrich), as described [19]. The gels and the blotted membranes were scanned by an optical scanner (8800F, Canon Inc.).

### Mechanical measurements

The calcium-activated force and the responses to stretch of the muscle fibers were measured in the activating solution, as described earlier [20]. Repetitive square pulses of stretch (2–3% fiber length, 5 or 10 Hz) were applied at the plateau of isometric calcium-activated force (pCa = 4.0, 20 °C), and the force and length signals were digitized by using a data acquisition system (USB-6210, National Instruments) for further analysis. The acquisition program was the one provided with the system.

## Additional files

Additional file 1: Figure S1. X-ray diffraction patterns of the flight and tymbal muscles of *Platypleura kaempferi.* (A) and (B), DLM; (C) and (D), basalar muscle; (E) and (F), tymbal muscle. A, C and E were recorded in the relaxed state, and B, D and E in rigor. (TIFF 7312 kb)

Additional file 2: Figure S2. X-ray diffraction patterns of the flight and tymbal muscles of *Terpnosia vacua.* (A) and (B), DLM; (C) and (D), tymbal muscle. A and C were recorded in the relaxed state, and B and D in rigor. (TIFF 4599 kb)

Additional file 3: Figure S3. X-ray diffraction patterns of the flight and tymbal muscles of *Graptopsaltria nigrofuscata.* (A) and (B), DLM; (C) and (D), basalar; (E) and (F), tymbal muscle. A, C and E were recorded in the relaxed state, and B, D and F in rigor. Weaker sampling on the layer line reflections may be due to the long-term storage (10 months in 50% glycerol). (TIFF 5668 kb)

Additional file 4: Figure S4. SDS gel electrophoretic and immunoblot patterns of the muscle fibers from *Graptopsaltria nigrofuscata.* (A), Coomassie brilliant blue-stained SDS gel electrophoretic pattern; (B), Western blot pattern obtained by using an antibody against flight muscle-specific troponin-I (troponin-H). Lanes: a, DLM; b, DVM; c, forewing basalar; d, forewing subalar; e, hindwing basalar; f, hindwing subalar; g, forewing 3Ax, h, hindwing 3Ax; i, tymbal; j, tensor; k; leg. (TIFF 980 kb)
