# Supplementary figures and images for: The tymbal muscle of cicada has flight muscle-type sarcomeric architecture and protein expression
Source: Zoological Lett. 2017 Sep 1;3:15. doi: 10.1186/s40851-017-0077-4 (PMC5581462; doi:10.1186/s40851-017-0077-4)

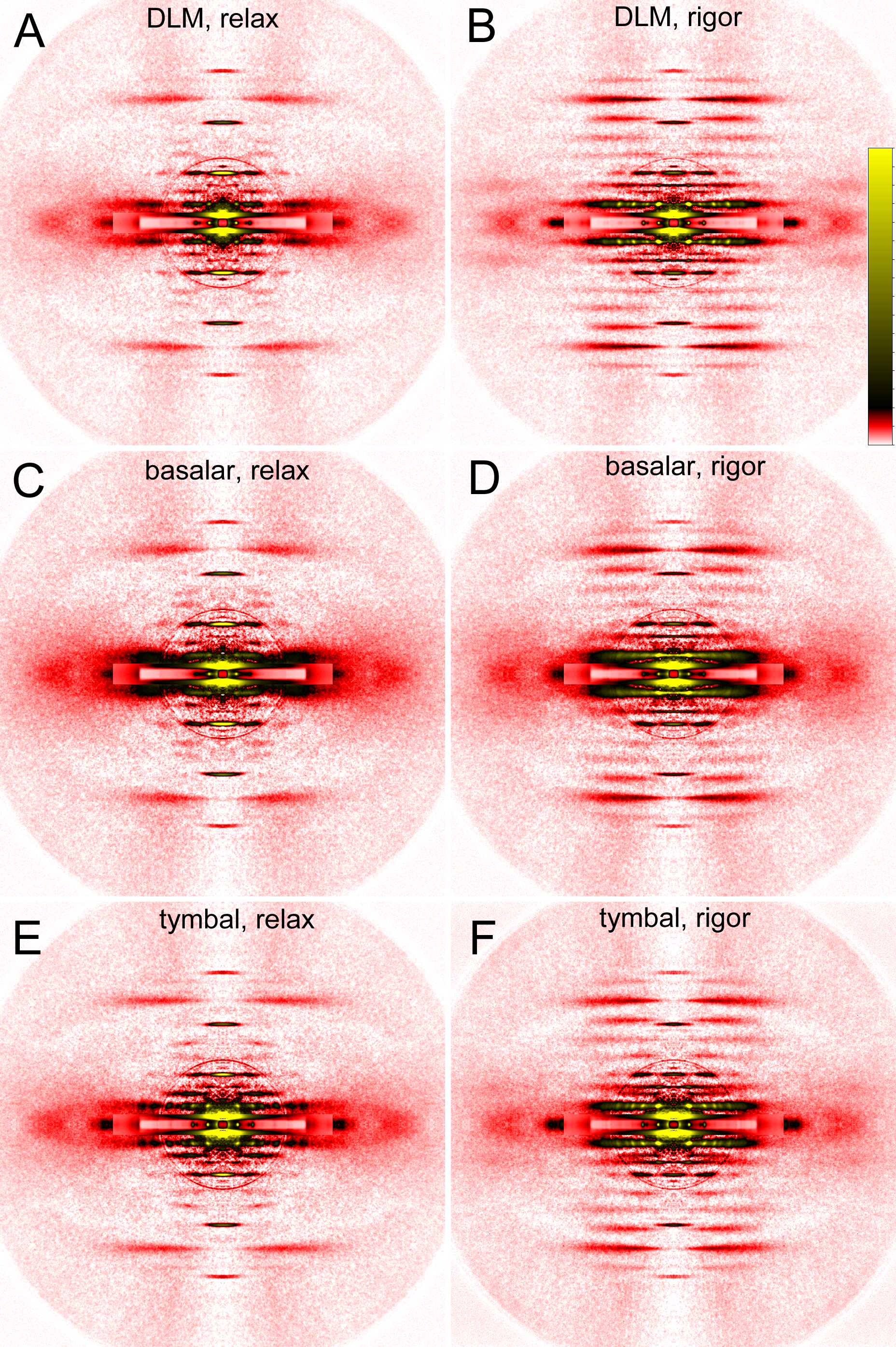

Supplement: Supplementary file 1 — X-ray diffraction patterns of the flight and tymbal muscles of Platypleura kaempferi. (A) and (B), DLM; (C) and (D), basalar muscle; (E) and (F), tymbal muscle. A, C and E were recorded in the relaxed state, and B, D and E in rigor. (TIFF 7312 kb) [file 40851_2017_77_MOESM1_ESM.tif]

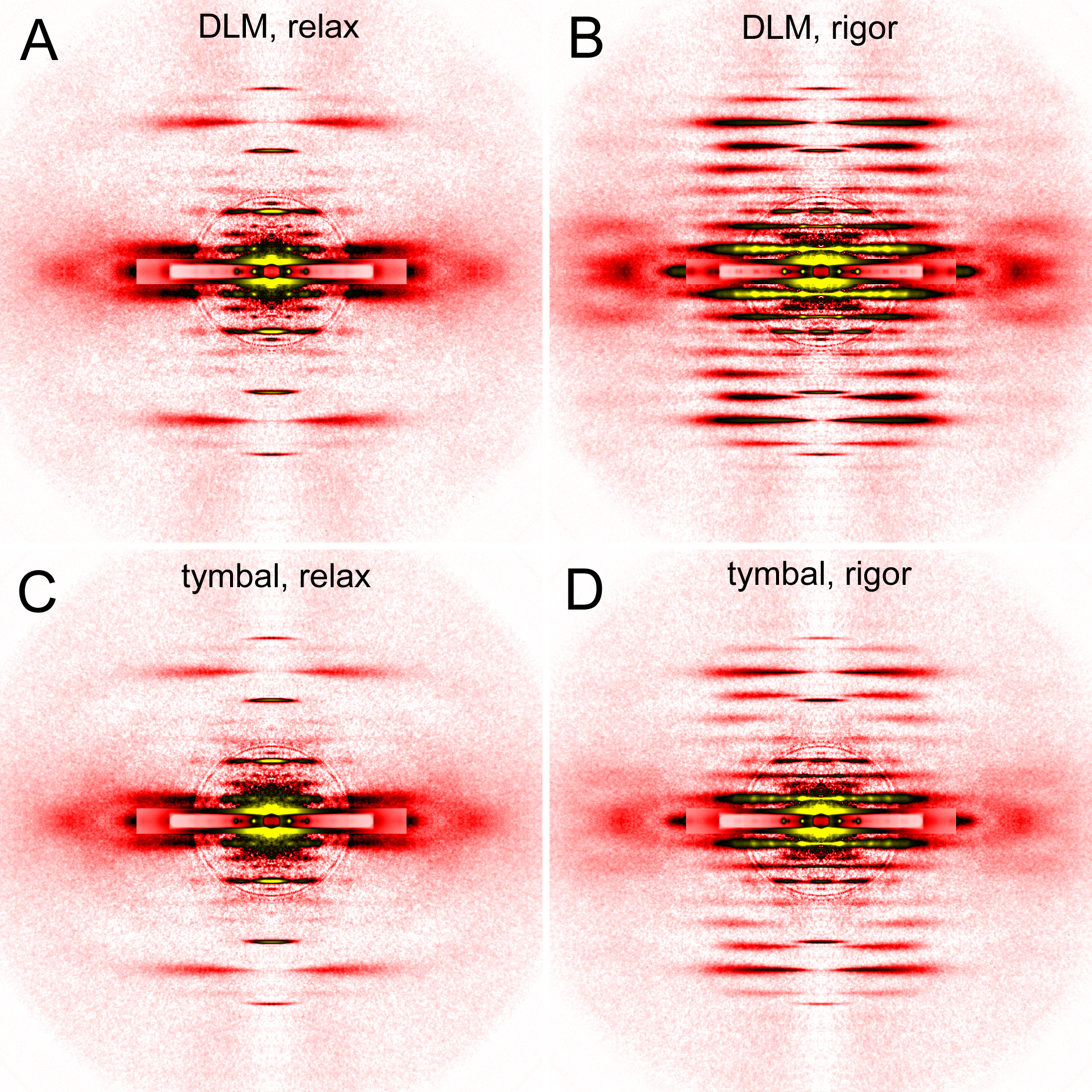

Supplement: Supplementary file 2 — X-ray diffraction patterns of the flight and tymbal muscles of Terpnosia vacua. (A) and (B), DLM; (C) and (D), tymbal muscle. A and C were recorded in the relaxed state, and B and D in rigor. (TIFF 4599 kb) [file 40851_2017_77_MOESM2_ESM.tif]

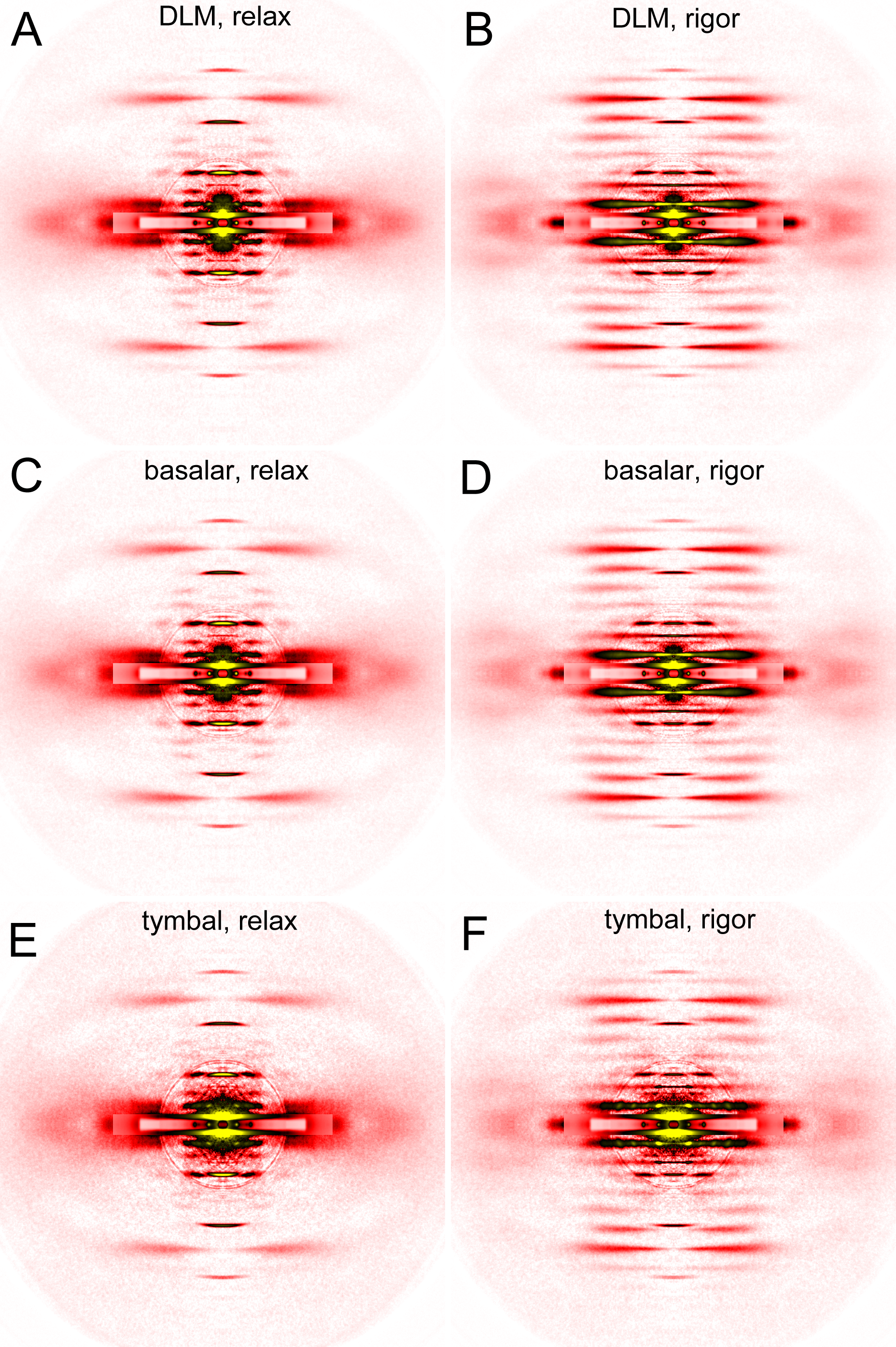

Supplement: Supplementary file 3 — X-ray diffraction patterns of the flight and tymbal muscles of Graptopsaltria nigrofuscata. (A) and (B), DLM; (C) and (D), basalar; (E) and (F), tymbal muscle. A, C and E were recorded in the relaxed state, and B, D and F in rigor. Weaker sampling on the layer line reflections may be due to the long-term storage (10 months in 50% glycerol). (TIFF 5668 kb) [file 40851_2017_77_MOESM3_ESM.tif]

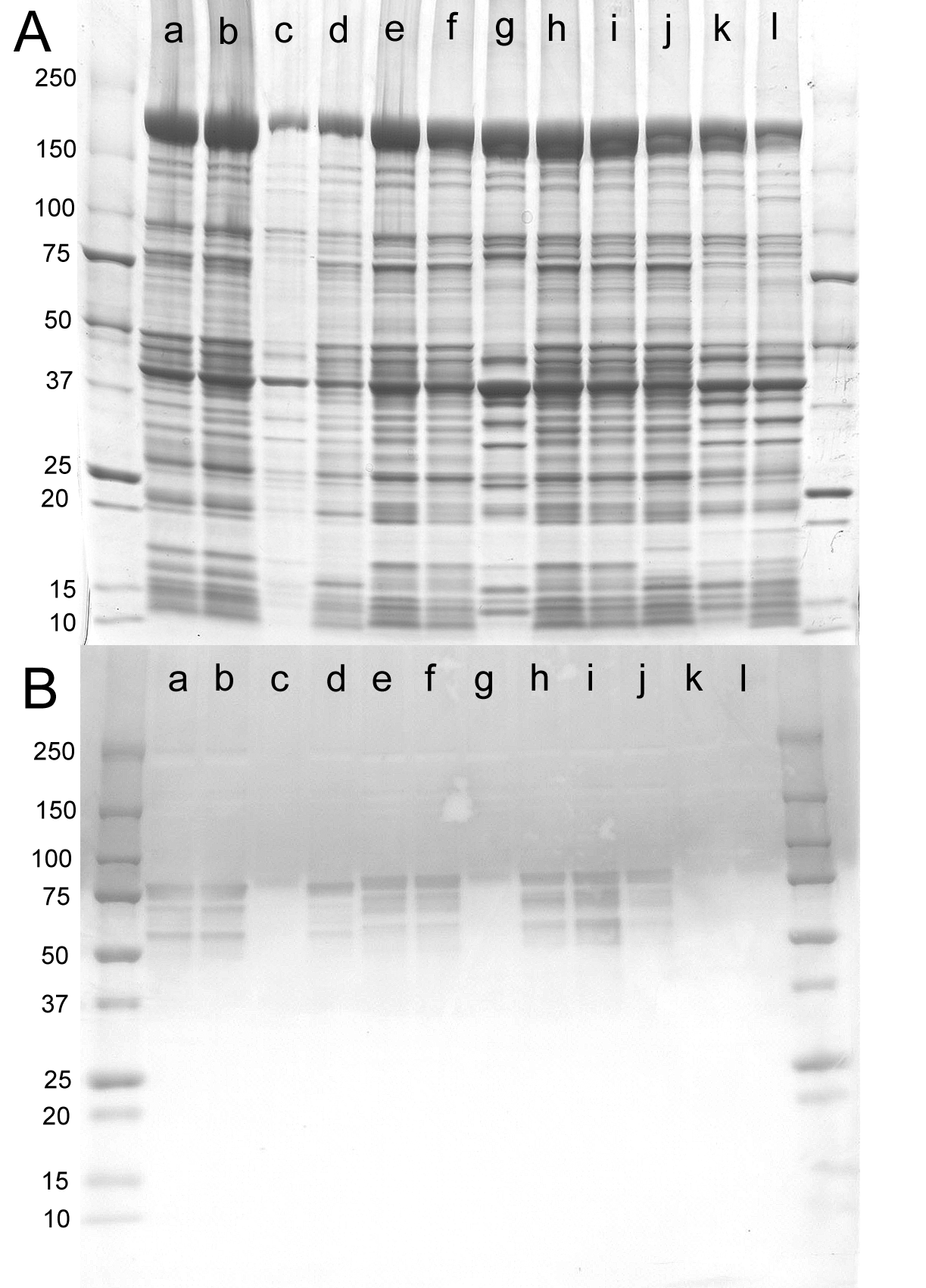

Supplement: Supplementary file 4 — SDS gel electrophoretic and immunoblot patterns of the muscle fibers from Graptopsaltria nigrofuscata. (A), Coomassie brilliant blue-stained SDS gel electrophoretic pattern; (B), Western blot pattern obtained by using an antibody against flight muscle-specific troponin-I (troponin-H). Lanes: a, DLM; b, DVM; c, forewing basalar; d, forewing subalar; e, hindwing basalar; f, hindwing subalar; g, forewing 3Ax, h, hindwing 3Ax; i, tymbal; j, tensor; k; leg. (TIFF 980 kb) [file 40851_2017_77_MOESM4_ESM.tif]
